# Vaccination process evaluation at COVID-19 vaccination centers in Lebanon: a national study

**DOI:** 10.1186/s40545-022-00459-3

**Published:** 2022-10-15

**Authors:** Abeer Zeitoun, Souheil Hallit, Maya Helali, Sirine Chehade, Carla Allam, Aya Ibrahim, Hani Dimassi, Rita Karam

**Affiliations:** 1 Quality Assurance of Pharmaceutical Products Department, National Pharmacovigilance Program, Lebanese Ministry of Public Health, Beirut, Lebanon; 2 School of Medicine and Medical Sciences, Holy Spirit University of Kaslik, P.O. Box 446, Jounieh, Lebanon; 3 Research Department, Psychiatric Hospital of the Cross, Jal Eddib, Lebanon; 4 School of Pharmacy, Lebanese American University, Koraytem, Beirut, 1102 2801 Lebanon; 5 Department of Chemistry and Biochemistry, Faculty of Sciences, Lebanese University, Beirut, Lebanon; 6 Pharmacology Department, Faculty of Medical Sciences, Lebanese University, Beirut, Lebanon

**Keywords:** Vaccination site, COVID vaccine, Pandemic, Lebanon

## Abstract

**Background:**

Upon the authorization of COVID-19 vaccines worldwide, national and international standards were developed to help integrate mass COVID-19 immunization campaigns into the healthcare network. The primary objective is to evaluate the overall COVID-19 vaccination process through on-site visits to vaccination centers all over Lebanon to assess whether these are abiding by the national and international requirements for both Pfizer-BioNTech and AstraZeneca vaccines. The secondary objective is to explore whether the type of the facility, private versus governmental, and educational versus non-education, affects the vaccination process in these centers.

**Methods:**

A convenient sample of 33 vaccination centers was selected from a list of all COVID-19 vaccination centers operating in Lebanon. Data were collected using a structured checklist developed following an extensive literature review of the national and international standards for Pfizer-BioNTech and AstraZeneca COVID-19 vaccines. A scoring system for the overall vaccination process was developed.

**Results:**

Quality deficiencies were identified in several steps of the immunization process; however, the international standards were followed in most vaccination centers visited despite their limited resources. It was noticed that there were no significant differences between private and governmental, between educational and non-educational, and between Pfizer and AstraZeneca vaccination centers; the 33 vaccination centers visited have scored above 75 on the entire process immunization against COVID-19 with *P*-values above 0.05 in all steps evaluated.

**Conclusion:**

An optimization of the immunization process should be performed to ensure that the practice is within international standards. This can be done by conducting periodic vaccination center visits, implementing clear guidelines, training staff involved in the vaccination process, and ensuring continuous support of the Lebanese Ministry of Public Health.

## Background

On January 30th, 2020*,* the World Health Organization (WHO) declared a public health emergency of international concern due to the outbreak of a Severe Acute Respiratory Syndrome Coronavirus 2 (SARS-CoV-2), also known as Coronavirus disease 2019 (COVID-19). By March 11th, 2020, the outbreak was declared a pandemic due to its rapid global spread [1]. One essential strategy to control this pandemic and allow all affected sectors including the economy, healthcare services, and quality of life to safely resume, was the rapid development of safe and effective vaccines [2]. Unprecedented efforts were made to develop vaccines against COVID-19 and develop them in large numbers simultaneously and in a short period of time. Both traditional and new technology platforms were adopted to develop these vaccines [3].

In a randomized controlled trial conducted in December 2020, a two-dose regimen of the messenger ribonucleic acid (mRNA) Pfizer-BioNTech (PZ) vaccine, showed 95% effectiveness in preventing COVID-19 infection [4]. The adenovirus-based COVID-19, known as the Oxford-AstraZeneca (AZ), also showed a 76% efficacy against symptomatic SARS-CoV-2 infection [5]. COVID-19 Vaccines were granted Emergency Use Authorization (EUA) in Lebanon early 2021, following authorization by the Food and Drug Administration (FDA) and the European Medicines Agency (EMA) [6–8]. In Lebanon, there are different types of health facilities. First, the healthcare system belongs to two sectors: the public sector that is fully managed and funded by the government, and the private sector which includes institutions that are owned by private individuals or groups of doctors [9, 10]. Second, these hospitals are divided into educational and non-educational facilities. Educational health facilities are medical centers that provide training and education to current and future health professionals in addition to delivering medical care to patients. Conversely, non-educational hospitals are not involved in graduate medical education [11].

The national vaccination campaign against COVID-19 started on February 14, 2021. Since then, around 5,563,105 doses have been administered, out of which 2,589,697 doses were the first of a series, 2,281,678 were second doses, 650,168 were third, and 41,562 already started with their fourth dose. VCs immunizing against COVID-19 were spread over Beirut, Mount Lebanon, Bekaa/Baalbeck-Hermel, South Lebanon, and North Lebanon. A pandemic of this scale has not occurred recently. Hence, a guide on “Lebanon National Deployment and Vaccination Plan for COVID-19 vaccines” was developed by the Ministry of Public Health (MoPH) and was published to simplify the understanding of the COVID-19 immunization process in local vaccination centers (VCs) [6]. International and national guidelines were used during the preparation of this manual to help detail all steps of the COVID-19 vaccination process; these include transportation, handling and storage, vaccine preparation and reconstitution, pre-vaccination assessment, vaccine administration, and post-vaccination process [11–17]. Two important documents were used in this manual, namely the “National Guidelines on Good Storage & Distribution Practices of Pharmaceutical Products in Lebanon” and the “Good Cold Chain Management for Temperature-Sensitive Pharmaceutical Products” [18, 19]. Indeed, it is the VCs’ duty to abide by these guidelines as stated in the Minister Decision N^o^ 334-2021 [20].

Gaps in the cold chain management process are one of the common factors limiting the equitable access to a successful immunization across countries [21]. The WHO describes vaccines as sensitive products that may be impaired when exposed to temperature excursions. This may lead to suboptimal therapeutic effect of the vaccines; if vaccines lose their potency, it cannot be restored [22, 23]. Therefore, continuous monitoring of vaccine is essential to ensure they are kept within the required temperature from production to administration.

Several essential activities should be performed by individuals attending an immunization center. Upon their arrival, they are asked to register, and confirm their personal information (full name, date of birth, phone number) and appointments. Next, they are asked to go through screening questions to ensure their eligibility to receive the vaccine. Finally, they are vaccinated, and after a monitoring period of 15–30 min, they are allowed to exit the site [11–17, 23].

On September 1st, 2021, several reports of vasovagal reactions were reported to the national pharmacovigilance (PV) department from more than one VC across Lebanon after the administration of the same lot number of PZ vaccine. Following this incident, actions were taken by the Lebanese PV program. At the MoPH, “the Preventive Medicine Department” conducts random visits to the vaccination sites to assess the preparation and handling of the vaccines. The memo 46-2021 was issued by the MoPH to facilitate regular inspections of the vaccination process. Furthermore, it enabled the national PV officers to go on-site to ensure that the complete COVID-19 vaccination process is operating according to national and international standards across all governorates in Lebanon [24, 25]. Based on the visits conducted, it was found that VCs welcomed on average around 480 vaccine recipients per day. As for their operating staff, some VCs had a full team in charge of around 10 personnel involved in the process while others had only two.

The primary objective of this study was to evaluate VCs immunizing against COVID-19 through on-site visits and assess whether they were abiding by the requirements of both PZ and AZ vaccines [11–17, 23]. The secondary objective was to explore whether the type of the facility affects the immunization practice in these centers.

## Methods

### Study design

This national descriptive study was conducted between September 15th and December 17th, 2021. A convenient sample of VCs was selected from a list of all COVID-19 VCs operating in all governorates of Lebanon. The list was retrieved from the Ministry of Public Health website [26]. VCs were classified according to the type of COVID-19 vaccine available at the facility (PZ or AZ vaccines), as well as the centers’ geographic distribution. From each governorate, 25% of each of the PZ and the AZ VCs were selected for on-site visit. According to the COVAX, the national platform where individuals in Lebanon should register to receive their vaccination, the selected VCs had an average of 480 vaccine recipients per day [26]. A letter of credentials was issued by the Ministry of Public Health [25], to facilitate conducting the present study, and was shared with all visited VCs along with an informed consent form to be signed. Individuals at the VC were not given a prior notice about the visit. A total of 33 VCs was visited during the period of this study by national pharmacovigilance officers.

### Data collection

Data were collected using a structured checklist (56 questions) developed following an extensive literature review of the national and international standards for vaccines’ transportation, handling and storage, preparation, pre-vaccination, administration, and post-vaccination processes for PZ and AZ vaccines (Table 1).

**Table 1:** International recommendation for Pfizer and AstraZeneca vaccines transportation, handling and storage, preparation, pre-vaccination, administration, and post-vaccination processes*

Recommendation for vaccine transportation	Reference
Vaccines should be transported using a portable vaccine refrigerator or qualified container designed to transport vaccines within the temperature range recommended by the manufacturers between + 2 °C and + 8 °C	[13]
Vaccines should be transported by following the manufacturer instructions for packing configuration and proper conditioning of coolants	[13]
The vaccine should be received in a good condition	[13]
Immediately upon arrival at the vaccination center, the time and minimum/maximum temperature must be recorded	[13]
The amount of vaccine transported should be limited to the amount needed for the workday	[13]
Total score for vaccine transportation = 5 points	
Recommendation for vaccine handling and storage	Reference
Upon arrival at the vaccination center, vaccines should be immediately unpacked and placed in proper storage equipment (i.e., a portable vaccine refrigerator or qualified container)	[13]
Vaccine should be stored in their original packaging until ready for administration. Loose vials or syringes may be exposed to unnecessary light, potentially reducing potency, and may be more difficult to track for expiration dates	[13, 27]
Upon arrival at the vaccination center, expiration dates of the vaccines should be checked that they had not expired	[13]
Vaccines should be stored within the manufacturer-recommended temperature range between + 2 °C and 8 °C	[13]
Vaccine temperature should be reviewed and documented a minimum of 2 times during each workday (preferably at the beginning and middle of an 8-h shift) to ensure they remain at correct temperatures (i.e., between + 2 °C and + 8 °C)	[13]
Vaccine temperature should be monitored using a digital data logger	[13]
A backup power source should be available to handle power outage	[27]
Avoid placing or storing any items other than vaccines, diluents, and water bottles inside the storage units	[27]
Between + 2 °C and + 8 °C, unpunctured Pfizer-BioNTech vials should be stored in the refrigerator for up to 1 month (31 days)	[12]
Unpunctured AstraZeneca vials should be stored in cold chain conditions of + 2 °C to + 8 °C for a maximum of 6 months	[16]
Sometimes unused vaccine and diluent doses, unopened vials, expired vials, and potentially compromised vaccine may be returned for credit, even if they must be discarded. Contact the vaccine manufacturer/distributor for vaccine-specific information	[27]
Total score for vaccine handling and storage = 11 points	
Recommendation for vaccine preparation	Reference
Perform hand hygiene before vaccine preparation and follow aseptic technique	[12]
Expiration dates of vaccines should be checked again during preparation, and only vaccines that have not expired should be administered	[13, 14]
All staff members who receive vaccine deliveries as well as those who handle or administer vaccines should be trained in vaccine-related practices and procedures	[27]
Vaccines should be prepared in a clean, designated medication area, away from any potentially contaminated items	[13]
Note the date and time the vaccine was prepared on the vial	[12]
Name of the vaccine and lot number should be documented	[13]
Once drawn up, vaccines should be kept in the recommended temperature range	[13]
Total score for vaccine preparation = 7 points	
Recommendation for Pfizer preparation	Reference
Vials should be held at room temperature for up to 2 h before mixing	[12]
Inject 1.8 mL 0.9% sodium chloride (normal saline, preservative-free) diluent into the vaccine vial	[12]
Using a new, sterile alcohol swab for each vial, wipe off the stoppers of the diluent	[12]
Use a 21-gauge (or narrower) needle to withdraw the diluent into a mixing syringe	[12]
Using a new, sterile alcohol prep pad for each vial, wipe off the stoppers of the vaccine vials	[12]
Inject 1.8 mL 0.9% sodium chloride (normal saline, preservative-free) diluent into the vaccine vial	[12]
Using the mixing syringe, remove 1.8 mL of air from the vaccine vial to equalize the pressure in the vaccine vial	[12]
Gently invert the vaccine vial 10 times before and after adding the diluent	[12]
Withdraw 0.3 mL of mixed vaccine into the syringe	[12]
Remove any significant air bubbles with the needle still in the vial to avoid loss of vaccine	[12]
Keep mixed vaccine between 2 °C and 25 °C. Administer within 6 h. Discard any unused vaccine after 6 h	[12]
Total score for Pfizer preparation = 11 points	
Recommendation for AstraZeneca preparation	Reference
The vaccine vial should not be shaken while inspecting it	[16]
The vaccine vial stopper should be cleansed with a single-use 70% alcohol swab	[16]
Using aseptic technique, equalize the pressure by injecting 0.5 mL of air into the vial then draw up 0.5 mL of the vaccine into the syringe	[15]
Any air bubbles should be removed prior to removing the needle from vial to avoid losing any vaccine dose	[15]
Doses should be used within one hour if stored at room temperature, or within six hours if stored at + 2 °C to + 8 °C	[16]
Total score for AstraZeneca preparation = 5 points	
Recommendation for pre-vaccination	Reference
Staff should administer the vaccines to the correct age groups	[13]
Physical distancing and enhanced infection control measures should be implemented	[28]
Review the history of allergic reaction(s)	[29]
Patients should be screened for contraindications and precautions for the specific vaccine(s) in use before receiving that vaccine(s)	[13]
Immunization history and vaccine schedule are checked to ensure appropriate vaccine administration	[29]
Total score for pre-vaccination = 5 points	
Recommendation for vaccine administration	Reference
Patient’s name and date of birth should be verified prior to vaccination	[13]
Staff should complete a training on vaccine administration	[29]
Staff are administering vaccines using the correct route: intramuscular injection in the deltoid muscle	[12, 13]
Staff should administer the correct dosage (volume) of vaccine	[13]
Staff should use proper hygiene techniques to clean hands before vaccine administration, between patients, and anytime hands become soiled	[13]
Needles should not be recapped before disposal	[13]
Used needles and syringes should be immediately placed in a sharps container following administration	[13]
If vaccine administration errors are observed, corrective action should be taken immediately	[13]
Total score for vaccine administration = 8 points	
Recommendation for post-vaccination	Reference
Patients should remain for observation at the vaccination center for for 15–30 min post-vaccination	[12, 15]
Provide patient with relevant post-vaccination advice	[16]
All vaccination providers at the site are certified in cardiopulmonary resuscitation (CPR), are familiar with the signs and symptoms of anaphylaxis, know their role in an emergency, and know the location of epinephrine and are trained in its indications and use	[13]
There is a designated area at the site for management of patients with urgent medical problems	[13]
Total score for post-vaccination = 4 points	

*Each recommendation gets 1 point

During the visits, national pharmacovigilance officers from the PV department at the MoPH, all of whom were clinical pharmacists, went on-site to observe the process from the minute the vaccine recipient reaches the site until they exit. Their role included observing several steps of the immunization process, interviewing the team in action including the head of the VC, and filling the checklist in hand. In other words, the aforementioned checklist was completed based on the visiting team’s observations of the vaccination process and/or interviews with the operating staff. Each visit lasted up to one hour. Arabic, the country’s official language, was used during the interviews to enhance the center of vaccination responsible’ comprehension. VCs were reassured of the anonymity and the confidentiality of the data collected during the visits.

### Scoring system for the vaccination process

A scoring system was used for the questions in common between the PZ and the AZ vaccination process. In other words, the “PZ preparation” and the “AZ preparation” recommendation sections were not included in the total score calculation. The system grants one point to each VC abiding by the national and international standards throughout each step of the vaccination process (Table 1). Based on these scores, percentages were calculated and were classified into three categories: below 50%, between 50 and 75%, and above 75%. Any VC with a score that is above 50% was considered compliant with the national and international recommendations concerning the vaccination process against COVID-19.

### Ethical statement

All participants were provided with clear and easy-to-understand information about the research project in order to allow them to make an informed and voluntary decision about whether or not to participate in this study. In accordance with the privacy rule of the Health Insurance Portability and Accountability Act and the declaration of Helsinki, a written consent form was signed by each study site that indicates its agreement in participating in this study.

### Statistical analysis

Data collected through the checklist were entered and analyzed using the Statistical Package for the Social Sciences (SPSS) program version 23. Categorical variables were compared using Pearson’s Chi-squared test and Fisher’s exact test. Continuous variables were compared using Student’s *t*-test and Mann–Whitney *U* test were used to analyze the score and their percentages. Statistical significance was set at *P* < 0.05.

### Disclaimer

This national study was not sponsored by any institution or entity.

### Disclaimer

This study is not sponsored by any institution or entity.

## Results

A total of 33 VCs (20 administering the PZ vaccine and 13 administering the AZ vaccine) from governorates all over Lebanon were visited during the period of this study (Table 2).

**Table 2:** Description of the visited vaccination centers

Classification criteria	Total VC Count	Visited VC Count (%)
Total	100	33 (33.0)
Type of vaccine		
PZ	55	20 (36.4)
AZ	45	13 (29.5)
Sector		
Private	60	24 (40.0)
Governmental	40	9 (22.5)
Facility type		
Educational	43	21 (48.9)
Non-educational	57	12 (21.0)
Governorate		
Beirut^o^	9	6 (66.7)
Mount Lebanon^^^	29	9 (31.0)
South Lebanon^¶^	23	7 (30.4)
North Lebanon^¶¶^	23	6 (26.0)
Bekaa/Baalbeck-Hermel^‡^	16	5 (31.25)

ºBeirut governorate includes VC in Beirut area

^^^Mount Lebanon governorate includes VC in Aley, Baabda, Chouf, Matn, Jbeil, Keserwan, and Baskinta

^¶^South Lebanon governorate includes VC in Jezzine, Saida, Tyre, Bint Jbeil, Hasbaya, and Marjeyoun

^¶¶^North Lebanon governorate includes VC in Batroun, Bcharreh, Koura, Minieh-Danniyeh, Tripoli, and Akkar

^‡^Bekaa governorate includes VC in Rashaya, West Bekaa, Zahleh, Baalbeck, and Hermel

### Scoring system of the vaccination process

All VCs, regardless of their sector or facility type, had a total score above 75% in the overall vaccination process. One PZ VC which was private and non-educational scored less than 50% in the vaccine preparation process. Four VCs (2 AZ and 2 PZ), 3 of which were private and non-educational, scored less than 50% in the pre-vaccination process (Tables 3, 4 and 5).

**Table 3:** Comparison of the Pfizer and AstraZeneca vaccination centers during the vaccination process*

Quartile	Range	Pfizer, N (%)	AstraZeneca, N (%)	*P*-value
Transportation	> 75	20 (100.0)	13 (100.0)	–
Handling and storage	50–75	2 (10.0)	1 (7.7)	1.000
	> 75	18 (90.0)	12 (92.3)	
Vaccine preparation	< 50	1 (5.0)	0	0.798
	50–75	3 (15.0)	3 (23.1)	
	> 75	16 (80.0)	10 (76.9)	
Pfizer preparation	50–75	4 (20.0)	–	–
	> 75	16 (80.0)	–	
AstraZeneca preparation	> 75	–	13 (100.0)	–
Pre-vaccination	< 50	2 (10.0)	2 (15.4)	0.413
	50–75	0 (0.0)	1 (7.7)	
	> 75	18 (90.0)	10 (76.9)	
Administration	> 75	20 (100.0)	13 (100.0)	–
Post-vaccination	50–75	3 (15.0)	3 (23.1)	0.659
	> 75	17 (85.0)	10 (76.9)	
Total	> 75	20 (100.0)	13 (100.0)	–

*Statistical tests used included Pearson’s Chi-squared test or Fisher’s exact test

**Table 4:** Comparison of the private and governmental vaccination centers during the vaccination process*

Quartile	Range	Private, *N* (%)	Governmental, *N* (%)	*P*-value
Transportation	> 75	24 (100.0)	9 (100.0)	–
Handling and storage	50–75	3 (12.5)	0	0.545
	> 75	21 (87.5)	9 (100.0)	
Vaccine preparation	< 50	1 (4.2)	0	1.000
	50–75	4 (16.7)	2 (22.2)	
	> 75	19 (79.2)	7 (77.8)	
Pfizer preparation	50–75	2 (15.4)	2 (28.6)	0.587
	> 75	11 (84.6)	5 (71.4)	
AstraZeneca preparation	> 75	11 (100.0)	2 (100.0)	–
Pre-vaccination	< 50	3 (12.5)	1 (11.1)	0.314
	50–75	0 (0.0)	1 (11.1)	
	> 75	21 (87.5)	7 (77.8)	
Administration	> 75	24 (100.0)	9 (100.0)	–
Post-vaccination	50–75	4 (16.7)	2 (22.2)	1.000
	> 75	20 (83.3)	7 (77.8)	
Total	> 75	24 (100.0)	9 (100.0)	–

*Statistical tests used included Pearson’s Chi-squared test or Fisher’s exact test

**Table 5:** Comparison of the educational and non-educational vaccination centers during the vaccination process*

Quartile	Range	Educational, *N* (%)	Non-educational, *N* (%)	*P*-value
Transportation	> 75	21 (100.0)	12 (100.0)	–
Handling and storage	50–75	3 (14.3)	0	0.284
	> 75	18 (85.7)	12 (100.0)	
Vaccine preparation	< 50	0	1 (8.3)	0.599
	50–75	4 (19.0)	2 (16.7)	
	> 75	17 (81.0)	9 (75.0)	
Pfizer preparation	50–75	2 (15.4)	2 (28.6)	0.587
	> 75	11 (84.6)	5 (71.4)	
AstraZeneca preparation	> 75	8 (100.0)	5 (100.0)	–
Pre-vaccination	< 50	1 (4.8)	3 (25.0)	0.186
	50–75	1 (4.8)	0	
	> 75	19 (90.5)	9 (75.0)	
Administration	> 75	21 (100.0)	12 (100.0)	–
Post-vaccination	50–75	3 (14.3)	3 (25.0)	0.643
	> 75	18 (85.7)	9 (75.0)	
Total	> 75	21 (100.0)	12 (100.0)	–

*Statistical tests used included Pearson’s Chi-squared test or Fisher’s exact test

There were no significant differences detected in the vaccination process between centers administering PZ vaccines and centers administering AZ vaccines (Table 6), between VCs in the private and governmental sectors (Tables 7), and between the VCs in the educational and non-educational facilities (Table 8).

**Table 6:** Comparison of the scores between Pfizer and AstraZeneca vaccines during the vaccination process*

Process	Scoring and percentages	Pfizer (*N* = 20)	AstraZeneca (*N* = 13)	*P*-value
Transportation	Score/5	5.0	5.0	–
	Percentage	100.0	100.0	
Handling and storage	Score/10	9.0 ± 1.3	8.9 ± 0.9	0.848
	Percentage	90.0 ± 12.5	89.2 ± 8.6	
Vaccine preparation	Score/7	6.3 ± 1.4	5.9 ± 1.0	0.478
	Percentage	89.3 ± 20.1	84.6 ± 14.8	
Pfizer preparation	Score/11	9.7 ± 1.8	–	–
	Percentage	87.7 ± 16.5	–	
AstraZeneca preparation	Score/5	–	4.7 ± 0.5	–
	Percentage	–	93.8 ± 9.6	
Pre-vaccination	Score/5	4.5 ± 0.9	3.9 ± 1.4	0.213
	Percentage	89.0 ± 18.9	78.5 ± 28.8	
Administration	Score/8	7.9 ± 0.3	8.0	0.253
	Percentage	98.8 ± 3.8	100.0	
Post-vaccination	Score/4	3.3 ± 0.7	3.2 ± 0.8	0.803
	Percentage	82.5 ± 18.3	80.8 ± 20.8	
Total score**	Score/39	35.9 ± 2.5	35.0 ± 2.3	0.305
	Percentage	92.0 ± 6.4	89.7 ± 5.9	

*The statistical test used was the Student’s *t*-test for all steps except for the post-vaccination step. Mann–Whitney *U* test was used for the post-vaccination step

**The total score excludes the score of the “Pfizer Preparation” and “AstraZeneca Preparation”, and only includes the common questions to all VCs

**Table 7:** Comparison of the scores between private and governmental sectors during the vaccination process*

Process	Scoring and percentages	Private (*N* = 24)	Governmental (*N* = 9)	*P*-value
Transportation	Score/5	5.0	5.0	–
	Percentage	100.0	100.0	
Handling and storage	Score/10	8.9 ± 1.3	9.2 ± 0.4	0.430
	Percentage	88.8 ± 12.6	92.2 ± 4.4	
Vaccine preparation	Score/7	6.04 ± 1.3	6.3 ± 1.3	0.565
	Percentage	86.3 ± 18.1	90.5 ± 18.9	
Pfizer preparation	Score/11	9.9 ± 1.9	9.1 ± 1.7	0.373
	Percentage	90.2 ± 17.2	83.1 ± 15.2	
AstraZeneca preparation	Score/5	4.7 ± 0.5	4.5 ± 0.7	0.561
	Percentage	94.5 ± 9.3	90.0 ± 14.1	
Pre-vaccination	Score/5	4.3 ± 1.2	4.1 ± 1.1	0.700
	Percentage	85.8 ± 24.7	82.2 ± 21.1	
Administration	Score/8	7.9 ± 0.3	8.0	0.387
	Percentage	99.0 ± 3.5	100.0	
Post-vaccination	Score/4	3.3 ± 0.8	3.2 ± 0.8	0.820
	Percentage	82.3 ± 18.8	80.6 ± 20.8	
Total score**	Score/39	35.4 ± 2.7	35.9 ± 1.8	0.626
	Percentage	90.8 ± 6.8	92.0 ± 4.5	

*The statistical test used was the Student’s *t*-test for all steps except for the post-vaccination step. Mann–Whitney *U* test was used for the post-vaccination step

**The total score excludes the score of the “Pfizer Preparation” and “AstraZeneca Preparation”, and only includes the common questions to all VCs

**Table 8:** Comparison of the scores between educational and non-educational facilities during the vaccination process*

Process	Scoring and percentages	Educational (*N* = 21)	Non-educational (*N* = 12)	*P*-value
Transportation	Score/5	5.0	5.0	–
	Percentage	100.0	100.0	
Handling and storage	Score/10	8.9 ± 1.2	9.2 ± 0.8	0.447
	Percentage	88.6 ± 12.4	91.7 ± 8.3	
Vaccine preparation	Score/7	6.3 ± 1.0	5.8 ± 1.6	0.209
	Percentage	90.5 ± 14.5	82.1 ± 22.9	
Pfizer preparation	Score/11	9.9 ± 1.6	9.1 ± 2.3	0.373
	Percentage	90.2 ± 14.1	83.1 ± 20.6	
AstraZeneca preparation	Score/5	4.9 ± 0.4	4.4 ± 0.5	0.081
	Percentage	97.5 ± 7.1	88.0 ± 11.0	
Pre-vaccination	Score/5	4.6 ± 0.8	3.7 ± 1.5	0.072
	Percentage	91.4 ± 16.2	73.3 ± 29.9	
Administration	Score/8	8.0 ± 0.2	7.9 ± 0.3	0.690
	Percentage	99.4 ± 2.7	99.0 ± 3.6	
Post-vaccination	Score/4	3.4 ± 0.7	3.0 ± 0.7	0.121
	Percentage	85.7 ± 18.7	75.0 ± 18.5	
Total score**	Score/39	36.1 ± 2.3	34.5 ± 2.4	0.060
	Percentage	92.7 ± 5.8	88.5 ± 6.2	

*The statistical test used was the Student’s t-test for all steps except for the post-vaccination step. Mann–Whitney U test was used for the post-vaccination step

**The total score excludes the score of the “Pfizer Preparation” and “AstraZeneca Preparation”, and only includes the common questions to all VCs

### Descriptive analysis of the vaccination process

#### Vaccine transportation

All VCs followed national and international guidelines regarding vaccine transportation (Table 9).

**Table 9:** Comparasion of the Pfizer and AstraZeneca vaccination centers during the transportation process*

Questions	PZ *N* = 20 (%)	AZ *N* = 13 (%)	Total vaccines *N* = 33 (%)	*P*-value
1. Vaccines are transported using a portable vaccine refrigerator or qualified container to transport vaccines				
Yes	19 (100.0)	13 (100.0)	32 (100.0)	–
2. Vaccines are transported by following the manufacturer instructions for packing configuration and proper conditioning of coolants				
Yes	19 (100.0)	13 (100.0)	32 (100.0)	–
3. The vaccines are received in a good condition				
Yes	19 (100.0)	13 (100.0)	32 (100.0)	–
4. Time of arrival of the vaccine is documented				
Yes	19 (100.0)	12 (100.0)	31 (100.0)	–
5. Amount of vaccine ordered is based on the number of patients registered				
Yes	20 (100.0)	13 (100.0)	33 (100.0)	–

*Statistical tests used included Pearson’s Chi-squared test or Fisher’s exact test

#### Vaccine handling and storage

There was one PZ VC (3.0%) which did not keep the vaccine vials inside their boxes. Out of the 33 visited VCs, 3 (1 PZ and 2 AZ) stored the vaccines at temperatures outside the manufacturer -recommended range (between + 2 °C to + 8 °C). The refrigerator’s temperature was monitored once daily in 9 VCs (27.3%). Moreover, 3 VCs (2 PZ and 1 AZ) did not have a temperature data logger or an alarm system to track the refrigerator’s temperature. There were other items stored within the same refrigerator as the vaccine vials in 15 VCs (45.4%). In one PZ VC (3.0%), the manufacturer/distributor was not contacted for expired or defective vaccines (Table 10).

**Table 10:** Comparasion of the Pfizer and AstraZeneca vaccination centers during the handling and storage process*

Questions	PZ *N* = 20 (%)	AZ *N* = 13 (%)	Total vaccines *N* = 33 (%)	*P*-value
1. Upon arrival at the VC, vaccines are immediately unpacked and placed in proper storage equipment (i.e., a portable vaccine refrigerator or qualified container)				
Yes	20 (100.0)	13 (100.0)	33 (100.0)	–
2. Vaccine vials are kept inside their boxes				
No	1 (5.0)	0 (0.0)	1 (3.0)	0.698
Yes	17 (85.0)	13 (100.0)	30 (90.9)	
Not observed	2 (10.0)	0 (0.0)	2 (6.1)	
3. Upon arrival at the VC, expiry dates of vaccines are checked				
Yes	20 (100.0)	13 (100.0)	33 (100.0)	–
4. Before preparation, vaccines are stored based on the manufacturer-recommended temperature range (between + 2 °C and + 8 °C)				
No	1 (5.0)	2 (15.3)	3 (9.1)	0.479
Yes	17 (85.0)	11 (84.6)	28 (84.8)	
Not observed	2 (10.0)	0 (0.0)	2 (6.1)	
5. How often is this temperature of the storing refrigerator monitored?				
Once daily	5 (25.0)	4 (30.8)	9 (27.3)	0.554
Twice daily	2 (10.0)	3 (23.1)	5 (15.2)	
Several times per day	13 (65.0)	6 (46.2)	19 (57.6)	
6. Temperature of the storing refrigerator is tracked via a temperature data logger or an alarm system				
No	2 (10.0)	1 (7.70)	3 (9.1)	1.0
Yes	18 (90.0)	12 (92.30)	30 (90.9)	
7. In case of power outage, there is backup power sources				
Yes	20 (100.0)	13 (100.0)	33 (100.0)	–
8. At the VC, there are no other items stored in the same refrigerator with vaccine vials				
No	5 (25.0)	7 (53.8)	15 (45.4)	0.329
Yes	12 (60.0)	5 (38.5)	14 (42.4)	
Not observed	3 (15.0)	1 (7.7)	4 (12.2)	
9. Un-reconstituted vials are stored between + 2 °C and + 8 °C in the refrigerator for up to 1 month (31 days)				
Yes	20 (100.0)	–	20 (100.0)	–
10. Unopened vials may be stored between + 2 °C and + 8 °C in the refrigerator for a maximum of 6 months				
Yes	–	13 (100.0)	13 (100.0)	–
11. In case of expired or defective vaccines and diluents, the manufacturer/Distributor is contacted				
No	1 (5.0)	0 (0.0)	1 (3.0)	1.0
Yes	19 (95)	13 (100.0)	32 (97.0)	

*Statistical tests used included Pearson’s Chi-squared test or Fisher’s exact test

#### Vaccine preparation

There were 2 VCs (1 PZ and 1 AZ) which did not prepare vaccines in a designated area away from any potentially contaminated items. The vial label and expiration date were not verified prior to drawing up the doses in 10 VCs (30.3%). In 2 VCs (6.1%), individuals preparing the vaccines were not qualified and well-trained. The time of vaccine preparation was not documented in 13 VCs (39.4%). Moreover, the vaccine name and lot number were not documented in 3 VCs (2 PZ and 1 AZ). There was one PZ VC which kept the prepared vaccine syringes for more than 6 h (Table 11).

**Table 11:** Comparasion of the Pfizer and AstraZeneca vaccination centers during the vaccine preparation process*

Questions	PZ *N* = 20 (%)	AZ *N* = 13 (%)	Total vaccines *N* = 33 (%)	*P*-value
1. Proper hygiene is followed during the preparation process				
No	1 (5.0)	1 (7.7)	2 (6.1)	0.84
Yes	15 (75.0)	8 (61.5)	23 (69.7)	
Not observed	4 (20.0)	4 (30.8)	8 (24.2)	
2. Double check vial label and expiration date prior to drawing up				
No	1 (5.0)	0 (0.0)	10 (30.3)	0.808
Yes	15 (75.0)	9 (69.2)	15 (45.5)	
Not observed	4 (20.0)	4 (30.8)	8 (24.2)	
3. Qualified and well-trained individuals are responsible for the vaccine preparation				
No	1 (5.0)	1 (7.7)	2 (6.1)	1.0
Yes	19 (95.0)	12 (92.3)	31 (93.9)	
4. Vaccines are being prepared in a designated area, away from any potentially contaminated items				
No	1 (5.0)	1 (7.7)	2 (6.1)	1.0
Yes	19 (95.0)	12 (92.3)	31 (93.9)	
5. Time of preparation is documented				
No	5 (25.0)	8 (61.5)	13 (39.4)	0.036*
Yes	15 (75.0)	5 (38.5)	20 (60.6)	
6. Name of the vaccine and lot number are documented				
No	2 (10.0)	1 (7.7)	3 (9.1)	1.0
Yes	18 (90.0)	12 (92.3)	30 (90.9)	
7. Once drawn up, vaccines are kept at room temperature (+ 2 °C to + 25 °C) up to 6 h				
No	1 (5.0)	0 (0.0)	1 (3.0)	1.0
Yes	19 (95.0)	13 (100.0)	32 (97.0)	

*Statistical tests used included Pearson’s Chi-squared test or Fisher’s exact test

#### Pfizer preparation

In 6 VCs (30.0%), the top of the vaccine vial was not cleaned with a single-use alcohol swab, and it was not gently inverted 10 times before and after dilution. The volume drawn from the diluted solution was not 0.3 mL in 2 VCs (10.0%). Furthermore, significant air bubbles were not removed from the syringe before the administration in 4 VCs (20.0%) (Table 12).

**Table 12:** Description of the Pfizer vaccination centers during the vaccine preparation process*

Questions	PZ *N* = 20 (%)
1. Vials are held at room temperature for up to 2 h before mixing	
No	1 (5.0)
Yes	15 (75.0)
Not observed	4 (20.0)
2. Diluent used (specify type and amount used: 1.8 mL of 0.9% sodium chloride)	
Yes	16 (80.0)
Not observed	4 (20.0)
3. Top of the 0.9% sodium chloride vial cleaned with a single-use alcohol swab	
No	3 (15.0)
Yes	13 (65.0)
Not observed	4 (20.0)
4. Needle 21 gauge or narrower recommended for dilution process	
Yes	16 (80.0)
Not observed	4 (20.0)
5. Top of the vaccine of vial cleaned with single-use alcohol swab prior to piercing it	
No	6 (30.0)
Yes	10 (50.0)
Not observed	4 (20.0)
6. 1.8 mL of diluent added into the vaccine vial	
No	1 (5.0)
Yes	15 (75.0)
Not observed	4 (20.0)
7. Before removing the needle from the vaccine vial, pressure in the vial equalized by withdrawing 1.8 mL of air into the empty diluent syringe	
No	1 (5.0)
Yes	15 (75.0)
Not observed	4 (20.0)
8. Vaccine vial gently inverted 10 times before and after dilution	
No	6 (30.0)
Yes	10 (50.0)
Not observed	4 (20.0)
9. Draw up 0.3 mL of the diluted solution into a new sterile syringe with a needle appropriate for intramuscular injection	
No	2 (10.0)
Yes	14 (70.0)
Not observed	4 (20.0)
10. Significant air bubbles are removed from the syringe while the needle is still in the vial of the reconstituted vaccine	
No	4 (20.0)
Yes	12 (60.0)
Not observed	4 (20.0)
11. Mixed vaccine kept between + 2 °C and + 25 °C and used within 6 h	
No	1 (5.0)
Yes	15 (75.0)
Not observed	4 (20.0)

*Statistical tests used included Pearson’s Chi-squared test or Fisher’s exact test

#### AstraZeneca preparation

The top of the vaccine vial was not cleaned with a single-use alcohol swab before piercing it in 3 VCs (23.1%) (Table 13).

**Table 13:** Description of the AstraZeneca vaccination centers during the vaccine preparation process*

Questions	AZ *N* = 13 (%)
1. Do not shake the vaccine while inspecting the vial	
Yes	9 (69.2)
Not observed	4 (30.8)
2. Top of the vaccine of vial cleaned with single-use alcohol swab prior to piercing it	
No	3 (23.1)
Yes	6 (46.1)
Not observed	4 (30.8)
3. Using aseptic technique, inject 0.5 mL of air into the vaccine vial and draw up a 0.5 mL dose of vaccine	
Yes	9 (69.2)
Not observed	4 (30.8)
4. Significant air bubbles are removed from the syringe while the needle is still in the vial	
Yes	9 (69.2)
Not observed	4 (30.8)
5. Prepared vaccines must be used within one hour at room temperature and 6 h at + 2 °C to + 8 °C	
Yes	9 (69.2)
Not observed	4 (30.8)

*Statistical tests used included Pearson’s Chi-squared test or Fisher’s exact test

#### Pre-vaccination

In 13 VCs (39.4%), the waiting room was congested with physical distancing not respected. In 4 VCs (12.1%), the history of allergy, vaccine contraindications and precautions were not reviewed. In addition, the immunization history and vaccine schedule were not checked to ensure appropriate vaccine administration in 3 VCs (9.1%) (Table 14).

**Table 14:** Comparasion of the Pfizer and AstraZeneca vaccination centers during the pre-vaccination process*

Questions	PZ *N* = 20 (%)	AZ *N* = 13 (%)	Total vaccines *N* = 33 (%)	*P*-value
1. Staff in administering the vaccine to the correct age groups				
No	1 (5.0)	0 (0.0)	1 (3.0)	1.0
Yes	19 (95.0)	13 (100.0)	32 (97.0)	
2. Waiting room is not congested, physical distancing and shelter from weather elements is observed				
No	6 (30.0)	7 (53.8)	13 (39.4)	0.171
Yes	14 (70.0)	6 (46.2)	20 (60.6)	
3. Review of allergy reaction is done				
No	2 (10.0)	2 (15.4)	4 (12.1)	1.0
Yes	18 (90.0)	11 (84.6)	29 (87.9)	
4. A process for screening for contraindications and precautions is in place				
No	1 (5.0)	3 (23.1)	4 (12.1)	0.276
Yes	19 (95.0)	10 (76.9)	29 (87.9)	
5. Immunization history and vaccine schedule is checked to ensure appropriate vaccine administration				
No	1 (5.0)	2 (15.4)	3 (9.1)	0.547
Yes	19 (95.0)	11 (84.6)	30 (90.9)	

*Statistical tests used included Pearson’s Chi-squared test or Fisher’s exact test

#### Vaccine administration

In one PZ VC (3.0%), the vaccines were not administered intramuscularly in the deltoid muscle. Needles were recapped before disposal in one PZ VC (3.0%) (Table 15).

**Table 15:** Comparasion of the Pfizer and AstraZeneca vaccination centers during the administration process*

Questions	PZ *N* = 20(%)	AZ *N* = 13(%)	Total vaccines *N* = 33(%)	*P*-value
1. Patient’s name and of birth are verified prior to vaccination				
Yes	20 (100.0)	13 (100.0)	33 (100.0)	–
2. Staff have received a training for vaccine administration				
Yes	20 (100.0)	13 (100.0)	33 (100.0)	–
3. Staff are administering vaccines using the correct route: intramuscular injection in the deltoid muscle				
No	1 (5.0)	0 (0.0)	1 (3.0)	1.0
Yes	19 (95.0)	13 (100.0)	32 (97.0)	
4. Staff are administering the correct dosage (volume) of vaccine				
Yes	20 (100.0)	13(100.0)	33 (100.0)	–
5. Hand and workplace hygiene are being respected				
Yes	20 (100.0)	12 (92.3)	32 (97.0)	0.394
Not observed	0 (0.0)	1 (7.7)	1 (3.0)	
6. Needles are not being recapped before disposal				
No	1 (5.0)	0 (0.0)	1 (3.0)	0.640
Yes	19 (95.0)	12 (92.3)	31 (93.9)	
Not observed	0 (0.0)	1 (7.7)	1 (3.0)	
7. Used needles and syringes are being immediately disposed in a sharp container following administration				
Yes	20 (100.0)	12 (92.3)	32 (97.0)	0.394
Not observed	0 (0.0)	1 (7.7)	1 (3.0)	
8. If vaccine administration errors are observed, corrective action is being taken immediately				
Yes	5 (100.0)	3 (100.0)	8 (100.0)	–

*Statistical tests used included Pearson’s Chi-squared test or Fisher’s exact test

#### Post-vaccination

Patients were not monitored for 15–30 min post-vaccination in 11 VCs (33.3%). Counseling and after-care instructions were not provided in 7 VCs (21.2%). There were not a well-equipped and designated area for the AEFI management in 6 VCs (18.2%) (Table 16).

**Table 16:** Comparasion of the Pfizer and AstraZeneca vaccination centers during the post-vaccination process*

Questions	PZ *N* = 20 (%)	AZ *N* = 13 (%)	Total vaccine *N* = 33 (%)	*P*-value
1. Patients are being monitored for 15–30 min post-vaccination				
No	6 (30.0)	5 (38.5)	11 (33.3)	0.614
Yes	14 (70.0)	8 (61.5)	22 (66.7)	
2. Counseling and after-care instructions are given at the vaccination site				
No	4 (20.0)	3 (23.1)	7 (21.2)	0.637
Yes	16 (80.0)	9 (69.2)	25 (75.8)	
Not observed	0 (0.0)	1 (7.70)	1 (3.0)	
3. Qualified medical team is available to manage any emergencies or serious adverse event following immunization (AEFI)				
Yes	20 (100.0)	13 (100.0)	33 (100.0)	–
4. There is a well-equipped designated area at the site for AEFI management				
No	4 (20.0)	2 (15.4)	6 (18.2)	1.0
Yes	16 (80.0)	11 (84.6)	27 (81.8)	

*Statistical tests used included Pearson’s Chi-squared test or Fisher’s exact test

## Discussion

The present study was carried out to evaluate operations and practices performed at the level of the Lebanese VCs in the context of COVID 19 vaccines mass campaign.

### Scoring system of the vaccination process

In this study, all 33 VCs whether educational, non-educations, private, governmental, PZ, or AZ VC, scored above 75% in the overall vaccination process. Hence, all are considered compliant with the national and international guidelines on the operation of immunizing against COVID-19. In addition, there was no difference in the quality of the vaccination process between the governmental and the private sectors. In contrast to what was observed in our study, a study conducted in Vietnam revealed the quality of services provided by private sectors were significantly poorer than that of public providers [30]. According to the WHO, studies comparing the service and the quality of vaccination practices among public and private sectors across countries are lacking. Although these studies are limited in number, some service quality deficiencies have been identified in the private sectors which is in line with our findings [31]. For example, Soeung et al. found that healthcare workers in private sectors in Cambodia demonstrated lack of knowledge of vaccine management practices [32]. Furthermore, a study by Aljunid and Zwi revealed that vaccines were not always stored at the required temperatures [33, 34]. It is worth noting that low- to middle-income countries struggle in maintaining quality standards in VCs due to limited financial and human resources [31, 35] (Table 4).

### Descriptive analysis of the vaccination process

When the management of cold chain fails, the effectiveness of vaccines is affected. Therefore, the success of vaccination programs depends not only on the percentages of vaccine efficacy but also on avoiding the break of the cold chain throughout the immunization process [36–38].

#### Vaccine transportation

In our study, all assessed VCs received their vaccines from distributors/manufacturers via a qualified vaccine carrier, a portable vaccine refrigerator respecting the manufacturer instructions for packing configuration and proper condition of coolants. Typical vaccine distribution systems in most countries are based on a four-tier structure specifically, national, regional, district, and health facilities/clinics [39, 40]. A research article by Yauba et al., published in 2017, stated that the safest method to transport vaccines was to send them directly to health facilities with “ready to use” cold storage. However, due to financial limitations in some countries, the Centers for Disease Control and Prevention (CDC) recommended using portable refrigerators with a device for temperature monitoring to transport vaccines, which is in accordance with our findings [41].

#### Vaccine handling and storage

Our study revealed that the majority of VCs followed the recommended temperature during vaccine storage with only 9.1% of the VCs had vaccines exposed to temperatures exceeding 8 °C which goes against the recommended temperature range (+ 2 °C to + 8 °C) where suboptimal temperature reached 10 °C. Non-compliant VCs explained that the temperature monitoring device available at their site is very sensitive which may have led to such temperature excursions. According to them, opening the fridge may greatly affect the temperature on the monitor. Interestingly though, our practice has been found to be better than other countries. In fact, a phenomenological study from Ethiopia demonstrated that only 63% of the health facilities had good cold chain practices [42]. Results from a cold chain adaptability study conducted in 2015 across Bangladesh during the introduction of inactivated polio vaccine, revealed that temperatures above 8 °C were noted in 13–22% of vaccine carriers [43]. In another research paper studying the exposure of vaccines to suboptimal temperatures in 10 states in India, they noticed that vaccines were stored at temperatures above 8 °C 14.7% of the time [44]. In addition, a cross-sectional pilot study revealed that in Saravan, temperature excursions reached 11.9 °C and vaccine loggers recorded temperatures above 8 °C more than 80% of their time in storage [45]. Another cross-sectional study by Yakum et al. conducted in the Cameroon found that 26.9% of vaccine refrigerators were exposed to temperatures higher than 8 °C [46]. It is critical to establish a secure cold chain management of the vaccines. Indeed, a review by Yu et al. claimed that in case of breaches in the cold chain go unnoticed before administration, vaccine products may be ineffective or harmful [47, 48]. To avoid breaching the cold chain, regular temperature checks and the use of reliable monitoring equipment are essential. In addition, setting the temperature alarm within 0.5 to 1 °C below the appropriate range helps address any alteration in temperatures before it exceeds the standard limits [49]. This is consistent with our results where 72.7% of the VCs do regular temperature monitoring of their refrigerators, and 90.9% of the VCs track the temperature via a data logger or an alarm system. Hibbs et al. analyzed reports of vaccines that were kept at suboptimal temperatures reported to the Vaccine Adverse Event Reporting System (VAERS) database from 2008 till 2012 and described breaks in the cold chain management. They noticed that lack of caution, equipment failure, and improper personnel training were cited as reasons behind the breakdown of cold chain management [50]. In light of the latter, it is extremely important to train the personnel in charge of the vaccination process [51]. A study by Andress et al. clearly stated which information should be trained by which healthcare professional. This was further elaborated whereby it was explained that information about the immunization plan should be delivered by public health experts, vaccination procedures have to be demonstrated by nurses, and information regarding storage and vaccine preparation should be taught by pharmacists [52].

Our results showed that most of the VCs kept vaccines inside their boxes. According to the CDC, storing vaccines outside their boxes may result in their exposure to light which can potentially reduce their potency [53]. Furthermore, the study revealed that 45.4% of the VCs had products stored next to the vaccines. This may be due to the fact that most of the VCs visited had their vaccine vials stored in their hospital pharmacies, which limits the space available from separating them from other medications and injections [54]. In comparison to our findings, better results were observed in a study by Thielmann et al. conducted in North Rhine-Westphalia, one of the largest federal states in Germany, in which only 21% of the VCs failed to store vaccines in a separate refrigerator [55]. It is true that the CDC has emphasized the importance of avoiding placing any items other than the vaccines and their corresponding diluents inside the storage unit [53]. However, if medications and other products were to be stored in the same refrigerator, they need to be clearly labeled and stored in containers separate from vaccines; they can even be stored on different shelves. This helps prevent medical errors and unnecessary confusion [53, 56]. Furthermore, our results found that 97% of visited VCs confirmed that in case of vaccine defects, the manufacturer and the Lebanese Ministry of Public Health were directly contacted. For example, one of the sites identified black particles in some of the PZ vaccine vials after dilution. These vials were separated from other vials for further investigation. Similarly, in Japan, several batches of the Moderna COVID-19 vaccines were put on hold after finding “foreign material” including black particles in the vials [57].

#### Vaccine preparation

To prevent contamination with potential microorganisms from the environment, it is essential to prepare vaccine injections using proper hand hygiene and in a clean specified area [58]. Regarding this matter, findings of our study revealed that 69.7% of the VCs visited were following proper hand and workplace hygiene. In addition, results showed that 30% of the PZ VCs, and 23.1% of the AZ VCs cleaned the top of the vaccine vials with single-use alcohol swabs before piercing it. A study by Simon et al. described 9 cases of pyrogenic abscesses following the administration of diphtheria and tetanus toxoids immunization in the United States. Their study demonstrated that the external surface of the vial stopper could have been contaminated with Group A Streptococcus (GAS) which resulted in contaminating the need inserted through this stopper. Hence, the importance of swabbing the top of the vials with 70% isopropyl alcohol to avoid potential infections [59].

Following withdrawal of both vaccines, air bubbles may be of concern. It has been observed that air bubbles were overlooked in 20% of the PZ VCs visited. According to the American Society of Hospital Pharmacist (ASHP), Institute for Safe Medication Practices (ISMP), and United States Pharmacopeia (USP), small air bubbles are not problematic and may be ignored. However, large air bubbles can decrease the volume of the vaccine, thus increasing the risk of under dosing. Therefore, it is recommended to double check syringes for large air bubbles and address them before administration [60, 61].

One observation made in PZ VCs showed that 2 of them (10%) did not draw the correct vaccine volume of 0.3 ml. Although the clinical impact of a lower dose of the vaccine is not well established, it is not recommended by the CDC Advisory Committee on Immunization Practices (ACIP) [62].

#### Pfizer preparation

Specifically concerning the reconstitution process of PZ vaccines, findings revealed that 30.0% of the VCs did not invert the vaccine vial either before or after dilution, out of which some used the shaking method. A translational study in UK VCs by Kudsiova et al. demonstrated that mishandling PZ COVID-19 vaccines, mainly shaking them, can compromise their stability. Indeed, it was found that the lipid formulation has released up to 50% of mRNA due to shaking. Consequently, the absence of free mRNA protection may impact the efficacy of the vaccine [63].

#### Pre-vaccination

During the pre-vaccination process, results of this study showed that 39.4% of all VCs visited were congested. Our results are in line with a research article by Tagoe et al. conducted in Ghana where stakeholders face challenges with small physical spaces at healthcare facilities which is leading to congestion. In addition to that, chaos and increased risk of COVID-19 infection resulted due to overcrowding at the VCs in India were highlighted [64]. Therefore, to minimize crowding and ensure physical distancing, floor markings, clear seating layout, and personnel to guide the flow of people should all be considered [65].

#### Vaccine administration

A remarkable finding in our study was that one PZ VC was not using the proper practice for intramuscular (IM) injection. This VC was injecting the vaccine too high in the arm. A study by Keers et al. identified several causes that may lead to administration errors. Causes included physical factors such as fatigue, sickness, or sleep deprivation, and mental factors such as nervousness, boredom, bad mood, or stress. Other factors stated were improper training, lack of experience, interruptions during the process, and heavy workload on the staff; chaotic and busy working environments were also contributing to administration errors [66].

According to Bancsi et al., injecting a vaccine too high in the deltoid muscle may result in Shoulder Injury Related to Vaccine Administration (SIRVA). In addition, SIRVA can result from a deep injection that goes through the bursa [67, 68]. To note, the Vaccine Injury Compensation Program (VICP) have identified 13 cases of SIRVA; 62% were following the influenza vaccine, 15% following each the tetanus–diphtheria (Td) and tetanus–diphtheria and acellular pertussis (Tdap), and 8% following the Human Papillomavirus (HPV) vaccine. Similarly to our results, 46% of the 13 cases were injected too high in the arm. One proposed mechanism of SIRVA is the accidental injection of the vaccine in the synovial tissues causing an immune-mediated inflammatory reaction [69]. ISMP states this is triggered by using the incorrect IM injection technique. Therefore, it is necessary to use proper injection techniques during the administration of IM vaccinations. Ways to prevent SIRVA includes have the person doing the injection in a sitting position next to the seated patient and to completely expose the shoulder to better locate the deltoid muscle [70]. Consequently, providing proper training and education will allow new members joining the vaccination team to have the required knowledge to safely administer vaccines [71].

In 2002, the WHO estimated that around 40% of Hepatitis B virus (HBV) and Hepatitis C virus (HCV) infections, and 2.5% of Human Immunodeficiency Virus (HIV) infections among Health Care Workers (HCW) are linked to occupational exposures to sharp objects and needles [72]. A study by Mehta et al. found that out of the 342 cases of needle stick injuries reported across hospitals in Mumbai, India, 66 cases were through trash bags, 35 cases were due to needle recapping and 17 cases were during needle disposal [73]. Our data showed that 93.9% of VCs did not recap the used needles, and sharp containers were present in almost all VCs (97.0%) which decrease the risk of infections due to needle stick injuries. According to the National Institute for Occupational Safety and Health, these infections can be avoided if healthcare professionals avoid the recapping of needles, dispose used needles directly into sharps containers, report any injury related to sharps for proper follow-up, and take part in blood borne pathogen and needle stick prevention trainings [74].

Moreover, VCs who reported errors took corrective actions following these errors. These included 24-h observation of the patient at the ER, hospital admission for further follow-up, consulting physician experts based on the patients age group, and communicating of the error to the manufacturer and the MoPH for more guidance.

#### Post-vaccination

The literature clearly states that following the receipt of COVID-19 vaccines, individuals should be monitored for at least 15 min for any adverse events [75, 76]. Results have shown that around 33.3% of VCs visited allowed vaccine recipients to leave the VC directly after receiving their injection or were monitored for less than 15 min. The inability of the centers to keep the recommended physical distance in monitoring areas may have led to this practice. This may have forced centers to use a shorter observation period after getting vaccinated to decrease the risk of COVID-19 infection. In fact, in April 2020, a statement on the time of observation after COVID-19 immunization was issued by the Australian Technical Advisory Group on Immunization (ATAGI). They stated that the standard 15-min monitoring period remains the ideal protocol to follow. Nevertheless, they advise that a post-vaccination monitoring period of at least 5 min may be enough in immunization clinics where social distancing is not feasible if vaccine recipients meet the set criteria [76]. It is advised by the CDC to monitor individuals with history of allergies to injectable or vaccines, or any history of anaphylactic shock for at least 30 min after receiving their COVID-19 vaccine [77]. Insights from the American College of Allergy, Asthma, and Immunology COVID-19 Task Force stated that 71% of the anaphylaxis reactions post-COVID-19 vaccines reported to the Vaccine Adverse Event Reporting System (VAERS) between December 14th and December 23rd 2021 occurred within 15 min of receiving the vaccines [78]. Consequently, screening is an essential step before vaccine administration to be able to assess the monitoring period required. Following our observation, 12.1% of the VCs in our study skipped the screening process and review of allergy history. The main reason behind this divergence is that vaccine recipients have already gone over the screening questions before receiving their first dose which eliminates the need to screen again. However, the CDC recommends that even if the same vaccine was given previously, patients should still be screened prior to vaccination. One reason is that screening questions may have been updated based on the newest recommendations. Another reason is that the health status of patients might have changed since the last time they received their dose [79].

### Limitations and strengths

There were several possible limitations that need to be addressed. Visiting all VCs across Lebanon was not feasible; therefore, a selection of a small sample size was necessary. To consider the sample size appropriate, it was estimated that in each governorate, 25% of the centers should be visited. This cutoff was not reachable in two governorates: Akkar and Nabatiyeh. Some steps of the vaccination process could not be observed mainly due to completed tasks prior to team arrival. Hence, some steps could not be assessed leading to indeterminate answers. This might have influenced the quality of the data obtained. Moreover, a potential reporting bias (a distortion of presented information from research due to the selective disclosure or withholding of information), from the officers involved in the sites evaluation, might be possible. Lastly, the Hawthorne effect was inevitable in this report. Indeed, it was noted that some healthcare professionals demonstrated suboptimal performance because of the awareness of being. Our study reflected the good practice of the Lebanese mass immunization campaigns against COVID-19 in more than third of the VCs in a country that is facing economic, social, and political crises. The extensive geographical coverage and the inclusion of public, private, educational, and non-educational healthcare facilities are considered points of strength. This ensured the inclusion of different socio-demographic and cultural characteristics. Moreover, to the best of our knowledge, this study is the first to assess the organization, implementation, and performance of mass immunization centers against COVID-19 in the Middle East/North Africa region.

## Conclusion

Mass immunization campaigns remains the best and fastest way for vaccines to reach the greatest number of individuals. The current study evaluates VCs all over Lebanon during vaccination against COVID-19. Quality deficiencies were identified in several steps of the immunization process. Indeed, the gap between theory and practice is not new, and following international guidelines and recommendations to the letter remains a challenge. Therefore, it is essential that the Lebanese MoPH collaborates with vaccination centers to try and optimize the immunization process against COVID-19 through periodic vaccination center visits, clear guidelines, focused trainings, and continuous support, hereby enhancing the public trust in the healthcare system. Differences between public and private health facilities were noticed throughout our study. However, future studies are essential to investigate the effect and magnitude of the type of health facilities on the immunization practice.

## Data Availability

Not applicable.
